# Successful Conservative Management of Spontaneous Antegrade Migration of Feeding Jejunostomy

**DOI:** 10.5005/jp-journals-10018-1219

**Published:** 2017-05-05

**Authors:** Gautham Krishnamurthy, Narendra Pandit, Harjeet Singh, Rajinder Singh

**Affiliations:** 1 Department of General Surgery, Postgraduate Institute of Medical Education & Research, Chandigarh, India

**Keywords:** Antegrade migration, Feeding, Jejunostomy.

## Abstract

Successful conservative management of spontaneous antegrade migration of feeding jejunostomy of a patient with dysphagia due to carcinoma of nasopharynx is reported.

**How to cite this article:** Krishnamurthy G, Pandit N, Singh H, Singh R. Successful Conservative Management of Spontaneous Antegrade Migration of Feeding Jejunostomy. Euroasian J Hepato-Gastroenterol 2017;7(1):84-86.

## INTRODUCTION

Feeding jejunostomy is one of the common means to establish enteral access for nutrition for those who cannot consume food by oral route.^1^ Various methods have been devised for the placement of the tube.^1,2^ The enteral route may be temporary or permanent based on the indication for which the procedure is done. The feeding jejunostomy tube has its own complications with mechanical blockage due to kinking and volvulus or peritoneal displacement or bowel perforation.^1,3^ External displacement rather than internal migration is more likely to occur. We describe a rare case of spontaneous antegrade migration of the enteral feeding tube managed conservatively.

**Fig. 1:**
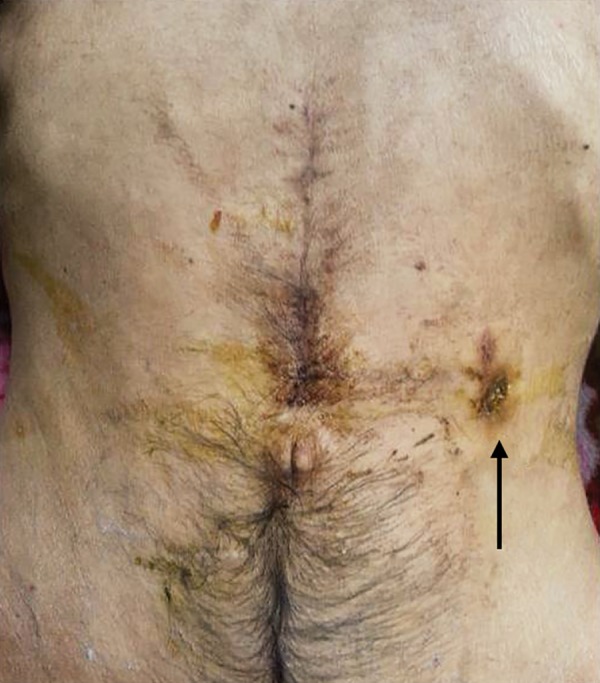
Jejunostomy site and healthy abdominal scar

### CASE REPORT

**Fig. 2:**
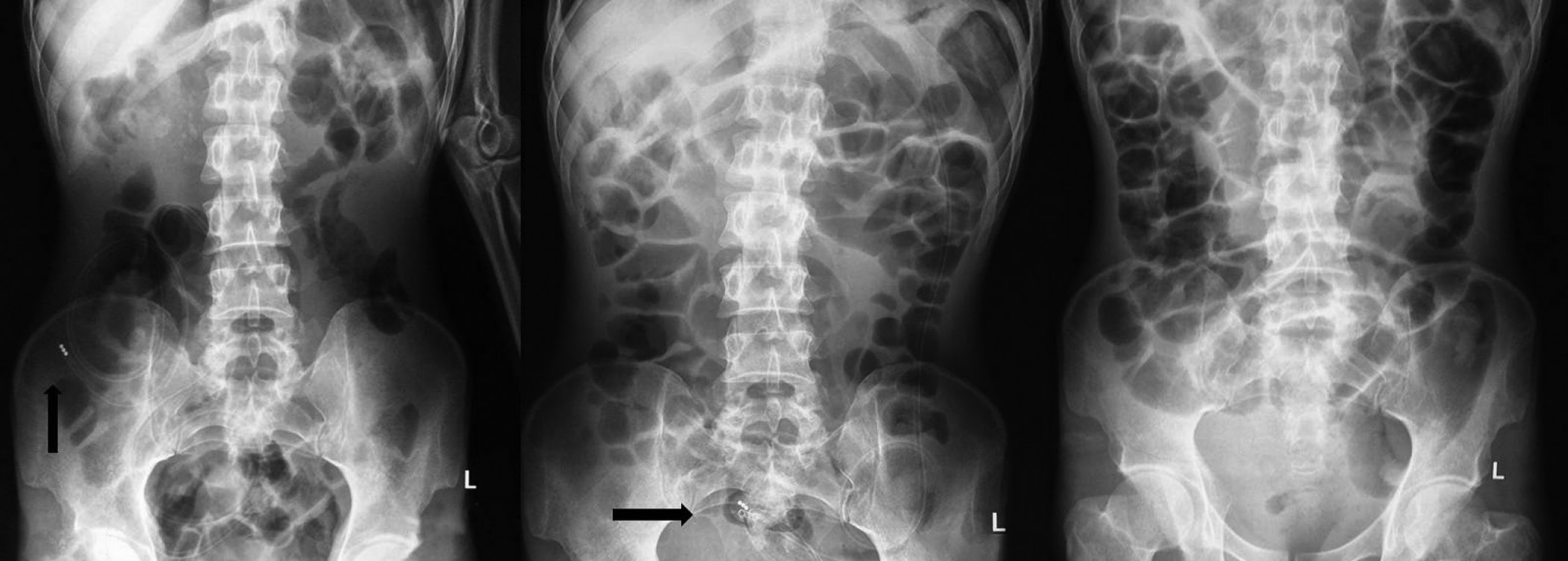
X-ray view of patient before discharge

A 40-year-old gentleman presented to the emergency department with complete internalization of the feeding jejunostomy for 1 day. Patient was evaluated for dyspha-gia 6 months earlier and was found to have carcinoma nasopharynx. In view of absolute dysphagia and failure to pass nasogastric tube, feeding jejunostomy was done using a 12F ryles tube. Patient was subjected to definitive chemoradiotherapy and dysphagia resolved. He was lost for follow-up thereafter. On examination, patient was hemodynamically stable and tolerating oral diet. Abdominal examination was soft with the feeding jejunostomy site and the abdominal scar healthy (Fig. 1). Per rectal examination was normal. Abdominal X-ray revealed migrated jejunostomy tube with the tip in the right iliac fossa. Expectant line of management was planned. Serial X-ray showed the antegrade movement of the jejunostomy tube. X-ray taken at 48 hours after migration showed the tube in rectum. The tube was removed per rectally. Patient was observed for 48 hours and discharged (Fig. 2). The patient was doing well on follow-up after 3 months.

### DISCUSSION

Nutrition is an essential component in the management of surgical patients and proper nutrition has been found to reduce complications in this population. Enteral nutrition is always preferred to parenteral nutrition, whenever feasible, given the septic complications and cost associated with parenteral nutrition.^4,5^

While gastrostomy and jejunostomy are the two common ways of establishing enteral access, the means of establishing access has evolved over the years, with open and laparoscopic methods being replaced by endoscopic and fluoroscopic means.^6^

Surgically placed jejunostomy is primarily done for enteral nutrition in patients undergoing major surgery of the upper digestive tract. Controversy still exists as to whether routine placement is required or jejunostomy needs to be performed only in selected patients.^7-9^ Patients who require prolonged nutritional supplementation because of neurological deficit in the mastication, deglutition, or the chewing processes also benefit from feeding jejunostomy. Apart from congenital malformations involving the upper gastrointestinal tract in pediatric patients, jejunostomy is also indicated in undernourished with unresectable or metastatic upper gastrointestinal malignancies causing obstruction that cannot be relieved by passage of nasoenteral tube or stenting.

Despite being a common and a useful option, it is not devoid of complications. The complications can be mechanical, infectious, gastrointestinal, and metabolic.^10^ Mechanical complications occur in approximately 2%, but variation has been noted with respect to the technique employed in performing the jejunostomy.^1^

Antegrade migration is a very rare complication of the feeding tube, with only case reports been reported.^11-14^ It can present with acute complication like perforation or obstruction or with features of malabsorption.^12^

The initial line of management of spontaneous migration is conservative provided patient does not have signs of acute abdomen.^13^ If there is a need for enteral access to be established, then a new feeding tube can be inserted through the previous site provided the tract has matured. In our case since the patient was tolerating complete solids, insertion of a new feeding line was not required. Serial abdominal radiographs would help in assessing the movement of the tube as well as complication due to migration, such as intestinal obstruction and perforation. Colonoscopic retrieval can be attempted if accessible.^14^ Surgical exploration is required if there is nonpropagation of the tube or occurrence of complication.

The peristaltic activity of small bowel makes the spontaneous antegrade migration of the feeding tube a definitive possibility and high index of suspicion is to be maintained. This preventable complication can be avoided by proper fixation of the tube to abdominal wall. Securing the redundant external portion in the form of circles also reduces the risk of internal migration. The free end of the feeding tube, when not in use, should be clamped with larger device or plug, such as a syringe. Patient education is an important component and they should be asked to report if there is visible reduction in the extra-abdominal part of the tube.

### CONCLUSION

Spontaneous antegrade migration of feeding jejunostomy is a rare complication. Expectant line of management can be followed in the absence of intestinal perforation or obstruction. Colonoscopic retrieval can be contemplated if the tube has migrated to the colon. Preventive measures of tube fixation and handling are essential to avoid this rare but potentially fatal complication.

## References

[1]  Tapia J, Murguia R, Garcia G, de los Monteros PE, Onate E (1999). Jejunostomy: techniques, indications, and complications.. World J Surg.

[2]  Young MT, Troung H, Gebhart A, Shih A, Nguyen NT (2016). Outcomes of laparoscopic feeding jejunostomy tube placement in 299 patients.. Surg Endosc.

[3]  Blumenstein I, Shastri YM, Stein J (2014). Gastroenteric tube feeding: techniques, problems and solutions.. World J Gastroenterol.

[4]  Braunschweig CL, Levy P, Sheean PM, Wang X (2001). Enteral compared with parenteral nutrition: a meta-analysis.. Am J Clin Nutr.

[5]  Pritchard C, Duffy S, Edington J, Pang F (2006). Enteral nutrition and oral nutrition supplements: a review of the economics literature.. JPEN J Parenter Enteral Nutr.

[6] ASGE Technology Committee,  Kwon RS, Banerjee S, Desilets D, Diehl DL, Farraye FA, Kaul V, Mamula P, Pedrosa MC, Rodriguez SA (2010). Enteral nutrition access devices.. Gastrointest Endosc.

[7]  Date RS, Clements WD, Gilliland R (2004). Feeding jejunostomy: is there enough evidence to justify its routine use?. Dig Surg.

[8]  Srinathan SK, Hamin T, Walter S, Tan AL, Unruh HW, Guyatt G (2013). Jejunostomy tube feeding in patients undergoing esophagectomy.. Can J Surg.

[9]  Gupta V (2009). Benefits versus risks: a prospective audit. Feeding jejunostomy during esophagectomy.. World J Surg.

[10]  Alivizatos V, Gavala V, Alexopoulos P, Apostolopoulos A, Bajrucevic S (2012). Feeding tube-related complications and problems in patients receiving long-term home enteral nutrition.. Indian J Palliat Care.

[11]  Bose AC, Shankar RR, Kate V, Ananthakrishnan N (2005). Spontaneous antegrade enteral migration of feeding jejunostomy tube.. Indian J Gastroenterol.

[12]  Prahlow JA, Barnard JJ (1998). Jejunostomy tube failure: malnutrition caused by intraluminal antegrade jejunostomy tube migration.. Arch Phys Med Rehabil.

[13]  Polychronidis A, Karayiannakis AJ, Perente S, Botaitis S, Simopoulos C (2003). Enteral migration of a Pezzer tube after a feeding jejunostomy: report of a case.. Surg Today.

[14]  Ozben V, Karata A, Atasoy D, Sim ek A,  Sarigul R, Tortum OB (2011). A rare complication of jejunostomy tube: enteral migration.. Turk J Gastroenterol.

